# Improvement of kynurenine aminotransferase-II inhibitors guided by mimicking sulfate esters

**DOI:** 10.1371/journal.pone.0196404

**Published:** 2018-04-24

**Authors:** Gayan S. Jayawickrama, Alireza Nematollahi, Guanchen Sun, William Bret Church

**Affiliations:** Group in Biomolecular Structure and Informatics, Faculty of Pharmacy, The University of Sydney, Sydney, New South Wales, Australia; Macquarie University, AUSTRALIA

## Abstract

The mammalian kynurenine aminotransferase (KAT) enzymes are a family of related isoforms that are pyridoxal 5’-phosphate-dependent, responsible for the irreversible transamination of kynurenine to kynurenic acid. Kynurenic acid is implicated in human diseases such as schizophrenia where it is found in elevated levels and consequently KAT-II, as the isoform predominantly responsible for kynurenic acid production in the brain, has been targeted for the development of specific inhibitors. One class of compounds that have also shown inhibitory activity towards the KAT enzymes are estrogens and their sulfate esters. Estradiol disulfate in particular is very strongly inhibitory and it appears that the 17-sulfate makes a significant contribution to its potency. The work here demonstrates that the effect of this moiety can be mirrored in existing KAT-II inhibitors, from the development of two novel inhibitors, JN-01 and JN-02. Both inhibitors were based on NS-1502 (IC_50_: 315 μM), but the deliberate placement of a sulfonamide group significantly improved the potency of JN-01 (IC_50_: 73.8 μM) and JN-02 (IC_50_: 112.8 μM) in comparison to the parent compound. This 3–4 fold increase in potency shows the potential of these moieties to be accommodated in the KAT-II active site and the effect they can have on improving inhibitors, and the environments in the KAT-II have been suitably modelled using docking calculations.

## Introduction

**Fig 1:**
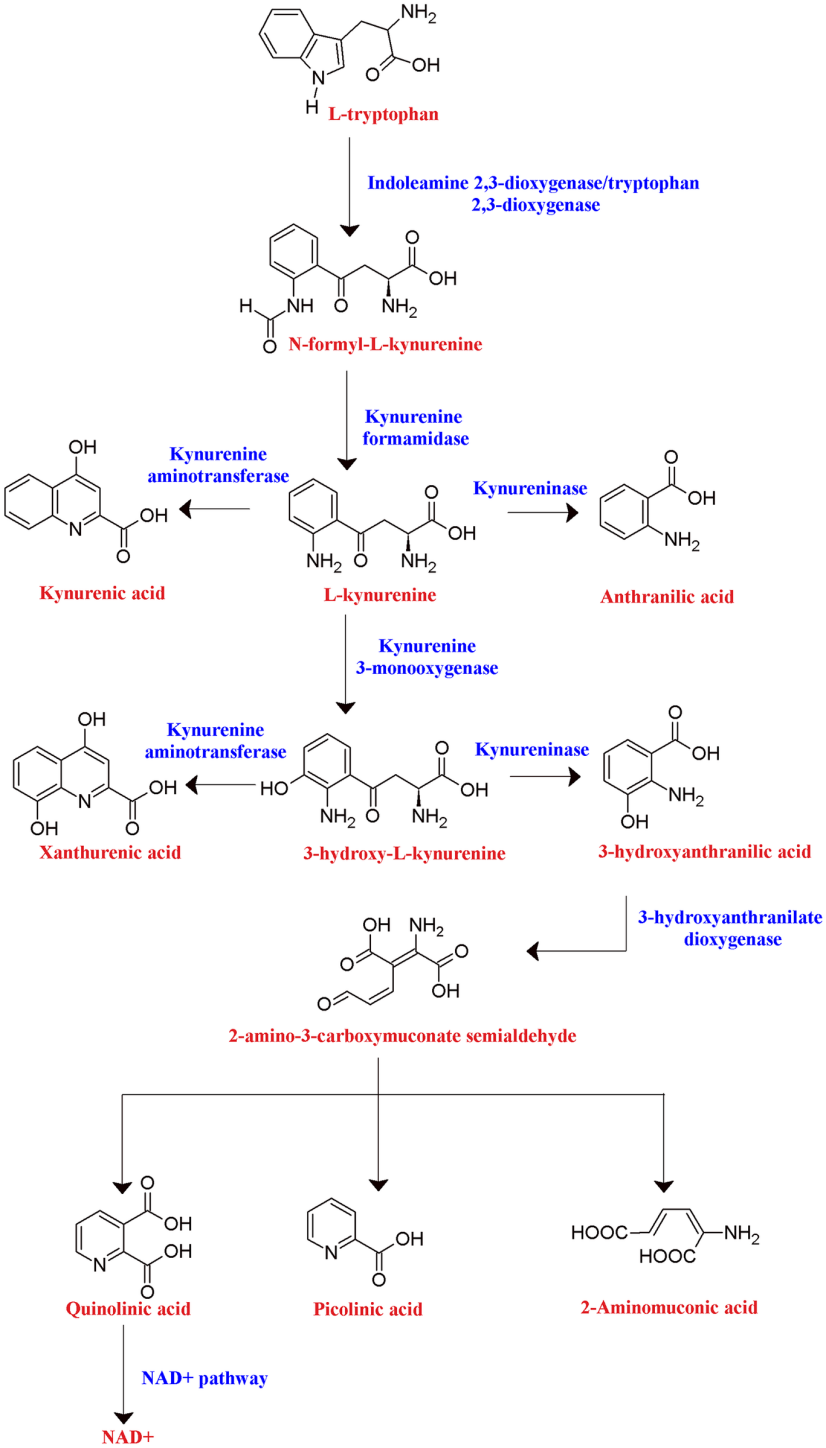
Tryptophan metabolism through the kynurenine pathway. Tryptophan metabolism proceeds through kynurenine in multiple branches, which includes the formation of KYNA by the KAT enzymes.

Kynurenic acid (KYNA) is a metabolite formed in the kynurenine pathway of tryptophan catabolism (Fig 1), produced when kynurenine is irreversibly transaminated into KYNA by the kynurenine aminotransferase (KAT) enzymes [1]. KYNA is an antagonist of the glycine and glutamate binding sites of NMDA receptors [2, 3], the α-amino-3-hydroxy-5-methyl-4-isoxazole propionic acid (AMPA) receptor [4], and kainate receptors [5]. By inhibiting the activity of these glutamatergic receptors and preventing excitotoxic attacks, as well as diverting the pathway from the formation of neurotoxic metabolites (such as 3-hydroxykynurenine and quinolinic acid), KYNA can be considered neuroprotective. KYNA also may have antagonistic effects on the α7-nicotonic acetylcholine receptors [6], which can play a role in glutamate and dopamine modulation, and also displays antioxidant [7] and anticonvulsive [8, 9] capabilities.

Despite this ability to be neuroprotective, elevated KYNA levels have been observed in the prefrontal cortex [10] and cerebrospinal fluid [11] of patients with schizophrenia, suggesting that a balance is necessary to prevent adverse events, particularly with regards to cognitive function. Animal studies have shown that lowering KYNA levels by application of KAT-II inhibitors increases glutamate [12, 13], acetylcholine [14], dopamine [15] and GABA [16] levels, for which roles in cognition have been established, and improves the performance of memory and spatial learning in rats and non-human primates [12, 13, 17]. A similar profile, with increased glutamate release and cognitive improvements, was also observed in KAT-II knock-out mice [18]. These pro-cognitive effects from KAT-II inhibition was neutralised by the administration of KYNA [13].

There are four isoforms of KAT, differing in structure and substrate specificities. KAT-I and KAT-III contain an aromatic crown present in the active site that enables the transamination of relatively hydrophobic substrates. In comparison, KAT-II and KAT-IV contain more neutral and polar amino acids, and KAT-II in particular is able to accommodate larger structures in the active site due to its flexible N-terminal region which helps construct the active site of the opposite subunit [19]. Individual KAT activity may also contrast greatly between species of animals, as for example the liver and kidney KAT activity of rats were significantly higher than the corresponding activity in cats [20].

The KAT isoforms are widely distributed in tissues, including the liver, kidney and brain [21]. KAT-II is thought to be the isoform predominantly responsible for the synthesis of KYNA in the brain, preferentially within astrocytes [22, 23]. KAT-I is relatively versatile with broader substrate recognition, making its activity less specific to KYNA synthesis [24]. By these considerations, KAT-II is targeted for the design of inhibitors to reduce brain KYNA levels. KAT-II is a homodimer of the fold type 1 family of pyridoxal 5’-phosphate (PLP)-dependent enzymes [25] and each of the subunits contain a large domain which has the PLP-binding site, a small domain containing the C-terminus, and an N-terminal arm [1]. The interface of the subunits houses an active site and the PLP-binding site is nearby for each subunit [1, 26].

PLP is covalently attached to Lys-263 of the enzyme by a Schiff base linkage [27]. When kynurenine enters the active site, this link is broken and a new aldimine bond is formed between PLP and the α-amine group of kynurenine [28]. This α-amino group is transferred onto the PLP, forming pyridoxamine phosphate (PMP), and KYNA is also produced from the remains of the substrate by spontaneous closure of its ring. PMP then transfers the α-amino group to an α-keto acid co-substrate to regenerate into the PLP form.

**Fig 2:**
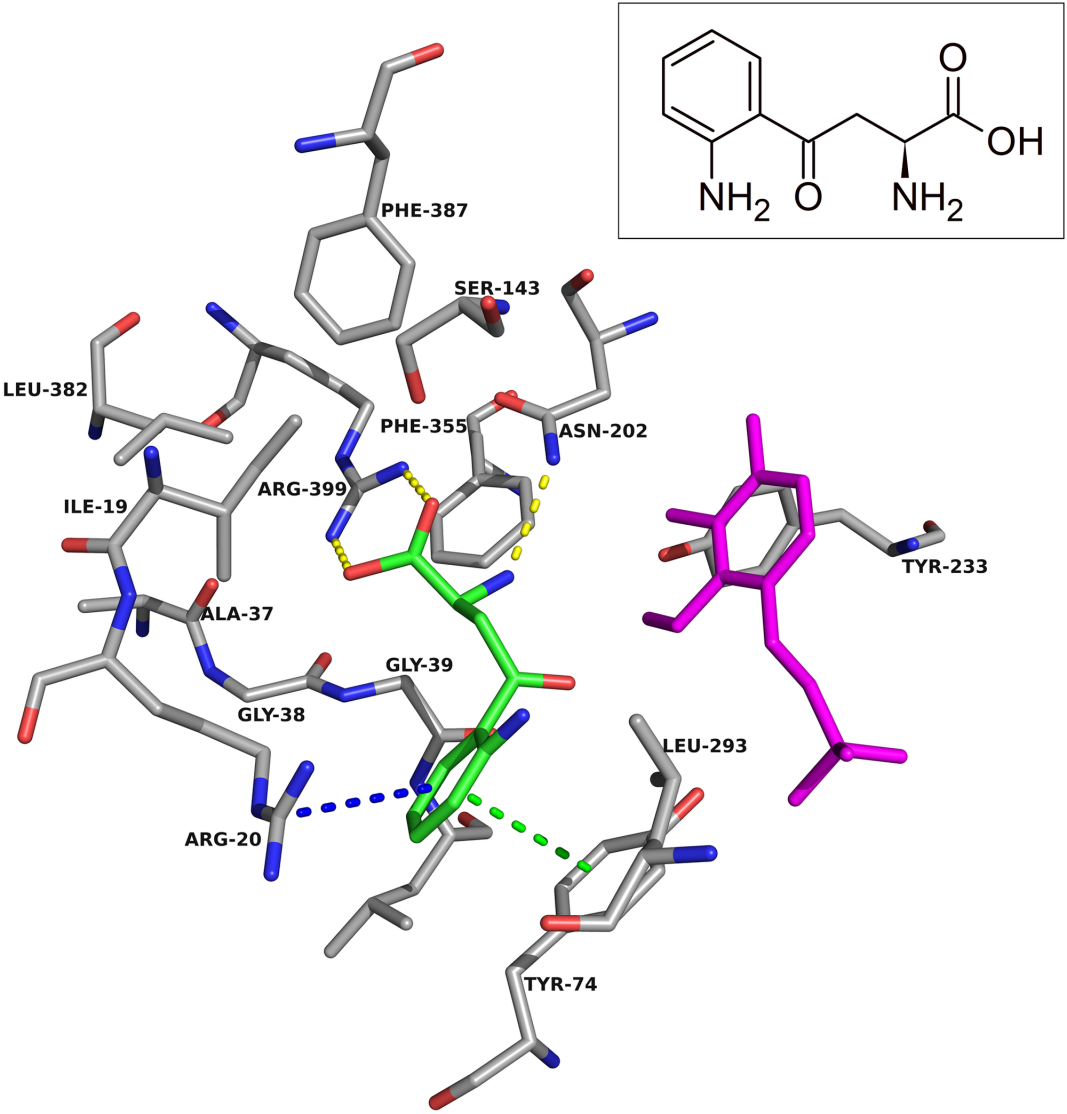
Kynurenine in the KAT-II active site. The amino acids within 5.0 Å of kynurenine (green) were chosen for display, with PLP in magenta and residue Tyr-142 removed for clarity. Depicted is the aromatic ring forming pi-pi interactions (green dashes) with Tyr-74 and pi-cation interactions (blue dashes) with Arg-20. A hydrogen bond (yellow dashes) between the amine group and Asn-202, and a salt bridge (yellow dashes) formed between the carboxyl and Arg-399 helps anchor kynurenine in the correct conformation for transamination. Image generated with PyMOL [29].

Arg-20 is another notable residue in the active site, with its side chain having pi-cation interactions with the aromatic ring of kynurenine (Fig 2) [26]. This region of the N-terminal residues allows the entry of the substrate into the active site by undergoing a conformational change. Asn-202 and Gly-39 forms hydrogen bonds with the amine group, and Arg-399 forms a salt bridge with the carboxyl group in kynurenine, and in doing so these residues help anchor the substrate in the necessary orientation within the active site [26].

Previous KAT-II inhibitors include S-ESBA, BFF-122, PF-04859989, BFF-816 and NS-1502 (S1 Fig). S-ESBA was designed by using kynurenine as a scaffold and adding an ethylsulfonyl substituent, which confers selectivity by increasing steric bulk and disqualifying it from occupying the relatively inflexible KAT-I [24]. The phenyl amino group was also removed from the scaffold, which eliminated the ability of KAT-II to transaminate it, rendering it inert and therefore inhibitory [30]. BFF-122, which shares a high similarity to the fluoroquinolone, levofloxacin, may also derive its selectivity from steric bulk that is accommodated by KAT-II [31]. Both BFF-122 and PF-04859989 feature heterocyclic ring systems and a primary amine group, the latter of which confers a mechanism of irreversible inhibition as it is observed to form a covalent adduct with PLP [31, 32]. NS-1502 combined features of these three inhibitors by incorporating a phthalimide core to resemble BFF-122 and PF-04859989, and adding an aromatic amino acid branch which resembled S-ESBA to improve the potency further [33].

Notably NS-1502 was designed to be reversible by the omission of the primary amine group present in BFF-122 and PF-04859989. Although irreversible inhibitors may be suitable in many instances, as PLP is a cofactor used by hundreds of enzymes, there is rationale in creating reversible inhibitors that specifically target KAT-II as opposed to PLP. This approach may be important to avoid the prospects of adverse events caused by depleting the cofactor or inhibiting off-target enzymes. Such concerns appear to be borne out by the use of carbidopa in patients with Parkinson’s disease, as it is able to permanently deactivate PLP, and is associated with the development of dyskinesias and increasing death rates [34, 35].

Steroid compounds, such as estrogen and sulfate esters of estrogen, have also been shown to inhibit a variety of PLP-dependent enzymes [36], including the KAT enzymes. Typically it has been reported that unconjugated steroids such as estradiol and estrone have a lower potency in inhibiting KAT from rat kidney in comparison to the sulfate ester forms [37]. The disulfate esters of estrogens, such as estradiol disulfate and diethylstilbestrol disulfate, were the most potent of these, while estrone sulfate was inhibitory at higher concentrations [37]. Similar results were found with phosphate esters, with estradiol diphosphate being very inhibitory towards rat kidney KAT [38]. The steroid phosphate compounds were also tested on another PLP-dependent enzyme, aspartate aminotransferase, with the phosphate groups appearing on different positions in the steroid structure. From this work, it was found that the phosphate group on the 17-position, as opposed to the 3-position, yielded the compound inhibiting with the greatest potency [38]. Similar results have been inferred in our lab, where a sulfate in the 17-position, instead of the 3-position, appears to increase the potency of the sulfated estrogen compounds inhibiting KAT-II [39]. From molecular docking, it is predicted that the increased potency may derive from the 17-sulfate forming hydrogen bonds with key residues in the active site, including, Asn-202 and Lys-263 [39].

In our current study, our goal was to use NS-1502 with a mind to improve its inhibitory capability further using detailed structural knowledge about the manner in which sulfated estrogens derive their potencies. Using an inhibition assay, we were able to evaluate the potency of two novel, reversible KAT II inhibitors, JN-01 and JN-02.

## Results and discussion

**Fig 3:**
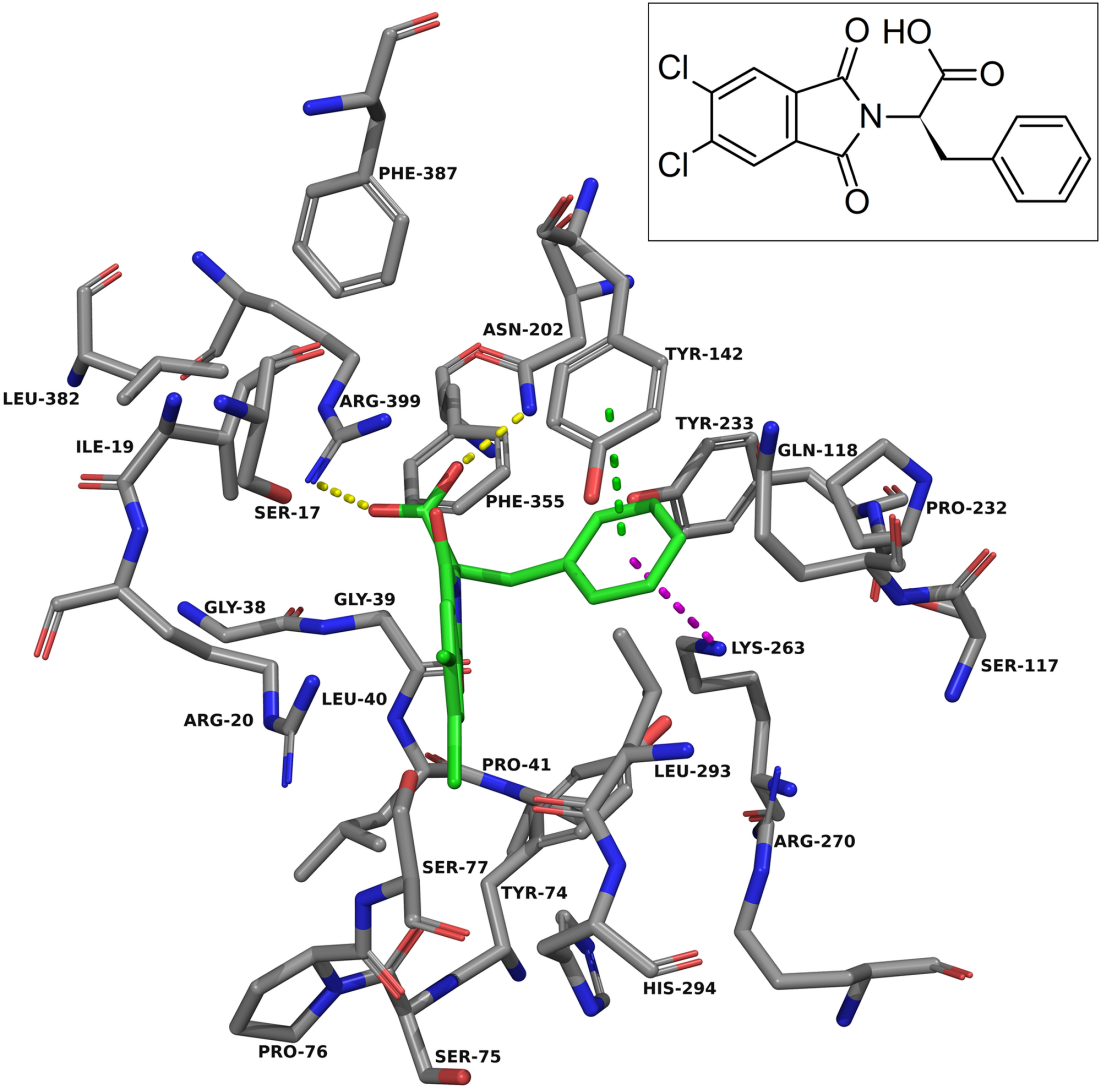
NS-1502 docked into the KAT-II active site. The amino acids within 5.0 Å of NS-1502 (green) were chosen for display. Residues Ser-143, Gly-144, and Gln-289 were removed for clarity. The carboxyl group forms hydrogen bonds (yellow dashes) with Asn-202 and Arg-399, and the aromatic ring has pi-pi interactions (green dashes) with Tyr-142 and pi-cation interactions (blue dashes) with Lys-263. Image generated with PyMOL [29].

**Fig 4:**
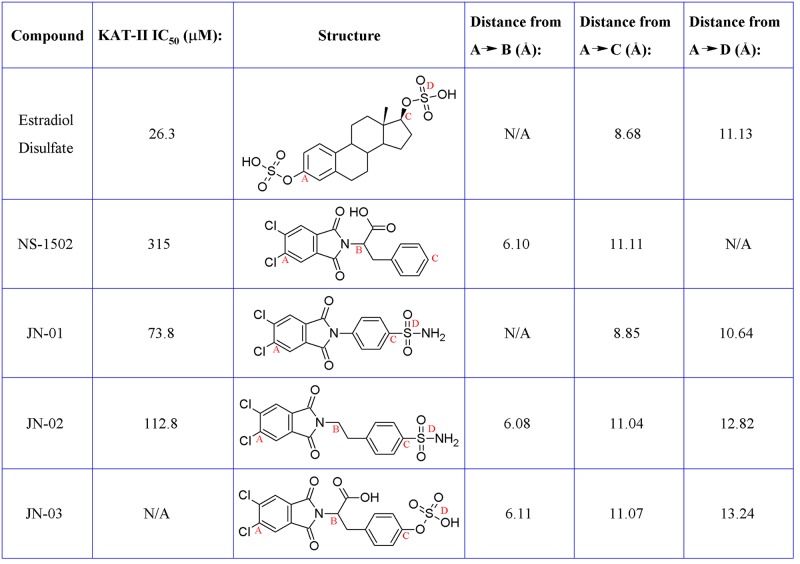
Comparative lengths of the structures when fully extended. Distances measured in the 3D conformer of the compounds, generated from LigPrep at the time of preparation for docking, from the first carbon atom (A) in each compound to the carbon from which the carboxyl branches off (B), the furthest carbon atom (C), or the sulfur atom in the sulfate/sulfonamide groups (D).

For the design approach to improving this inhibitor, we took the core of NS-1502 and strategically mimicked sulfated estrogens when synthesising JN-01. Due to the considerations from the inhibitor synthesis protocol, the sulfate moiety was replaced by a sulphonamide group, and the carboxyl present in NS-1502 was omitted. Evidence suggested that in the inhibitor estradiol disulfate, the sulfate on the 17-position, located on the end opposite to its aromatic ring (S2 Fig), was most important for its potency. Molecular docking predicted that the phthalimide core of NS-1502 (Fig 3) corresponded to the location of the aromatic ring of both kynurenine (Fig 2) and estradiol disulfate (S3 Fig). Therefore in designing JN-01, the rationale was to introduce a sulfonamide group onto the end opposite to the phthalimide core of NS-1502, so that it occupies the same location as the 17-sulfate of estradiol disulfate, where it can similarly exploit interactions with important nearby residues such as Asn-202 and Lys-263. Overall, JN-01 was also designed to ensure that it retained a length comparable to estradiol disulfate (Fig 4), with the difference coming from the relative sizes of the sulfonamide and the sulfate group.

JN-02 was designed with a similar concept in mind to JN-01, however the length of the compound was further increased, by an ethyl chain, which better reflected the size of NS-1502, while still incorporating the other features of JN-01 and estradiol disulfate. Although it is unquestionably larger than estradiol disulfate, JN-02 is flexible and can be accommodated to interact with similar active site residues.

The inhibition assay was previously performed on KAT-II with racemic NS-1502, BFF-122, and PF-04859989, which determined their IC_50_ to be 315 μM, 15–20 μM and 1–3 μM, respectively [33]. By varying the concentration of PLP in the assay conditions, NS-1502 was found to be reversible. When PLP concentration was increased, the inhibition caused by NS-1502 decreased as it competed with it. This contrasts with the irreversible inhibitor PF-04859989, which displayed similar inhibition as PLP concentration changed [33].

**Fig 5:**
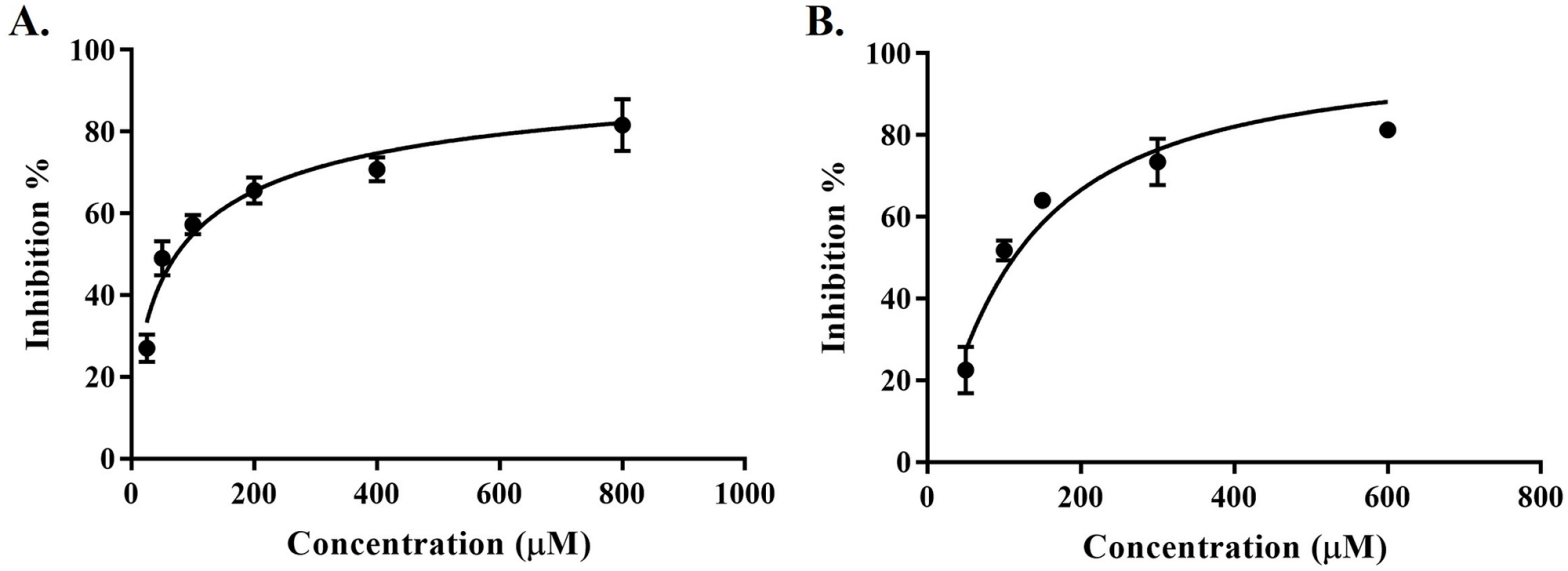
Dose-dependent inhibition of KAT-II. Inhibition assays with 0.5 μg KAT-II incubated for 10 min at 37 °C in a 50 μL reaction mixture containing 50 μM PLP, 5 mM α-ketoglutarate, 5 mM l-kynurenine and the inhibitor studied. The reaction was terminated with 0.8 M formic acid and analysed using HPLC. Experiments were performed as independent replicates, and figures were produced using GraphPad Prism v7.02 [40] **(A)** JN-01 inhibition (IC_50_: 73.8 (95% CI: 66.3–81.8) μM, R^2^: 0.91). **(B)** JN-02 inhibition (IC_50_: 112.8 (95% CI: 100.8–126.1) μM, R^2^: 0.92).

Relative to NS-1502, JN-01 and JN-02 displayed improved potency towards inhibiting KAT-II, with an IC_50_ of 73.8 μM (Fig 5A) and 112.8 μM (Fig 5B), respectively. The IC_50_ of both inhibitors for KAT-I in this assay is beyond the maximal concentration tested (2 mM), therefore they are considered to be selective towards KAT-II. Additionally, both inhibitors were found to be reversible. This reaffirms what was seen previously with estradiol disulfate and estradiol diphosphate where the 17- sulfate or 17-phosphate groups improved the inhibitory potency of the molecule, as these moieties were mirrored in JN-01 and JN-02 with the use of a sulfonamide group and similarly improved inhibition greatly from the parent compound, NS-1502.

Estradiol disulfate and estradiol has also been tested using this assay in these conditions and inhibits KAT-II with an IC_50_ of 26.3 μM and >2 mM, respectively [39]. The apparent increase in potency for KAT-II inhibition is 100-fold comparing estradiol disulfate and estradiol, whereas it is approximately 3–4 times for JN-01 and JN-02 in comparison to NS-1502. The absolute value for the IC_50_ is also lower for estradiol disulfate than it is for JN-01 and JN-02, suggesting that there also remains ample area for improvement in these new inhibitors. Two such areas that have been identified includes the lack of the carboxyl that was present in NS-1502 but not the new inhibitors, and also the sulfate present in estradiol disulfate being substituted by a sulfonamide moiety in the new inhibitors. The reintroduction of the carboxyl group and the replacement of the sulfonamide with the sulfate could maximise the number and strength of the hydrogen bonds formed with the residues in the active site. Molecular docking of NS-1502, JN-01, JN-02, and NS-1502 with a sulfate (JN-03) reinforces this.

**Fig 6:**
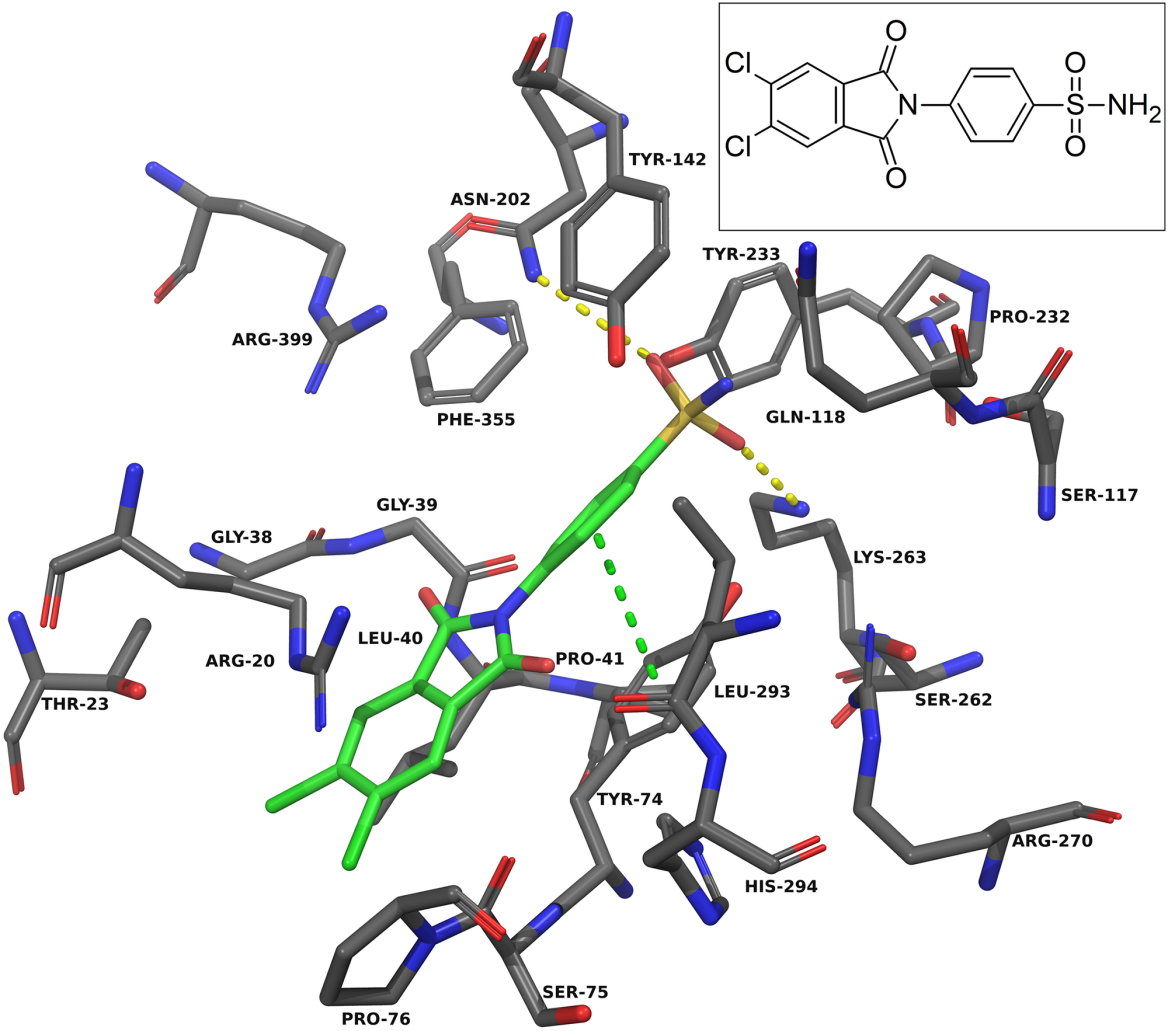
JN-01 docked into the KAT-II active site. The amino acids within 5.0 Å of JN-01 (green) were chosen for display. Residues Ser-143, Gly-144, and Gln-289 were removed for clarity. The oxygen atoms in the sulfonamide form hydrogen bonds (yellow dashes) with Asn-202 and Lys-263, and the aromatic ring has pi-pi interactions (green dashes) with Tyr-74. Image generated with PyMOL [29].

**Fig 7:**
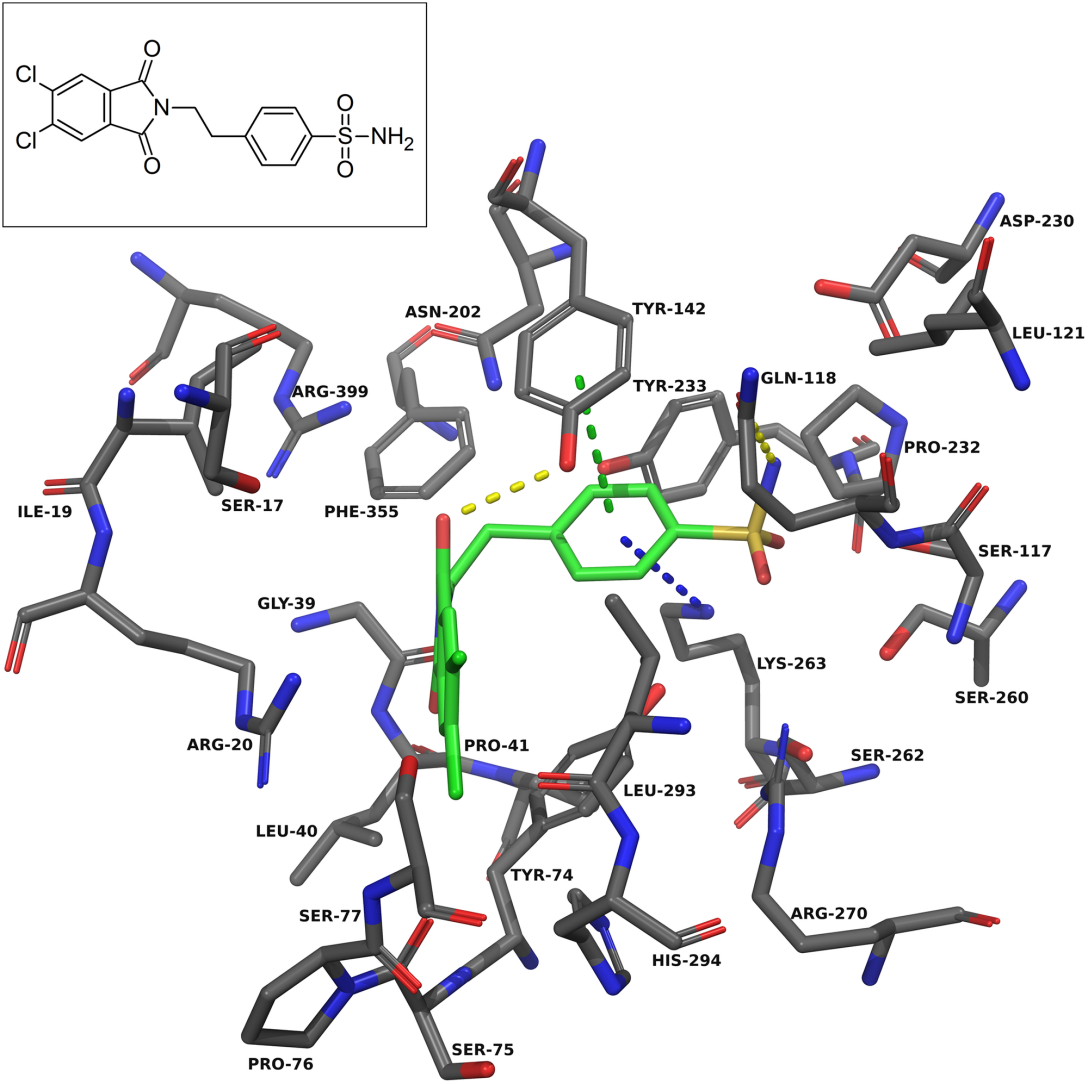
JN-02 docked into the KAT-II active site. The amino acids within 5.0 Å of JN-02 (green) were chosen for display. Residues Ser-143, Gly-144, and Gln-289 were removed for clarity. The amine in the sulfonamide forms hydrogen bonds (yellow dashes) with Gln-118, and the carbonyl in the phthalimide rings forms a hydrogen bond with Tyr-142. The aromatic ring has pi-pi interactions (green dashes) with Tyr-142 and pi-cation interactions (blue dashes) with Lys-263. Image generated with PyMOL [29].

Docking into KAT-II suggests that the best binding pose for NS-1502 has the phthalimide core situated near Arg-20, with the carboxyl forming hydrogen bonds with Arg-399 and Asn-202, and the lone six-membered aromatic ring forming pi-pi interactions with Tyr-142 and pi-cation interactions with Lys-263 (Fig 3). When JN-01 is docked, the phthalimide is orientated in a similar location, and the sulfonamide group has hydrogen bonding interactions with Asn-202 and Lys-263 (Fig 6). The whole of JN-02 docks in a similar pose to NS-1502, while lacking the carboxyl interactions but gaining an additional hydrogen bond between the sulfonamide and Gln-118 (Fig 7). Both JN-01 and JN-02 displayed the potential of this binding pocket, near Lys-263, in accommodating the sulfonamide moieties.

**Fig 8:**
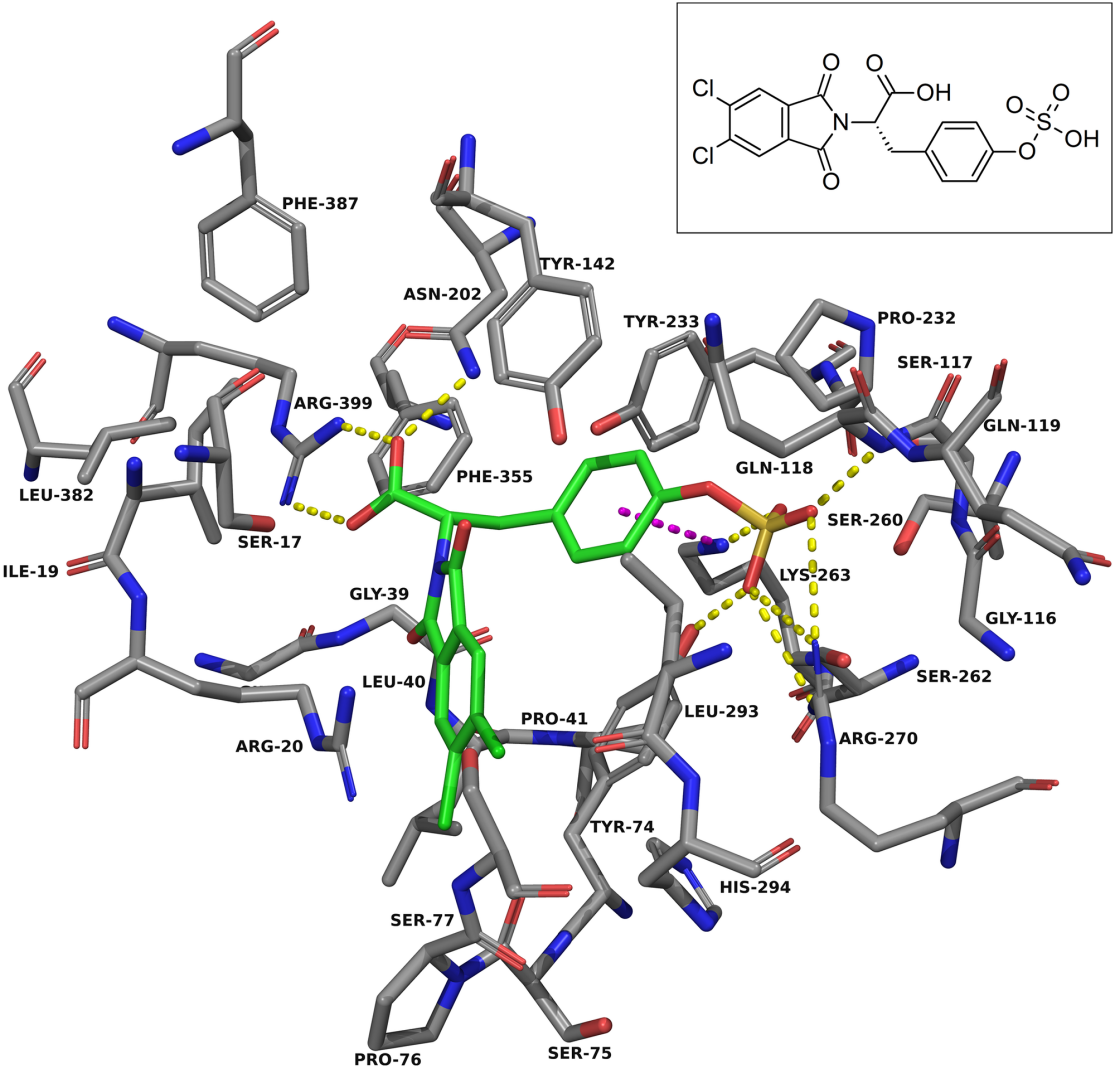
JN-03 docked into the KAT-II active site. The amino acids within 5.0 Å of JN-03 (green) were chosen for display. Residues Ser-143, Gly-144, and Gln-289 were removed for clarity. The sulfate forms hydrogen bonds with Tyr-74, Gln-118, Lys-263, and Arg-270, and the carboxyl moiety forms hydrogen bonds with Asn-202 and Arg-399. The aromatic ring has pi-cation interactions (blue dashes) with Lys-263. Image generated with PyMOL [29].

Considering NS-1502 and estradiol disulfate, the key interactions these two inhibitors have with the active site are with Arg-399, Asn-202 and Lys-263. A carboxyl on JN-02, branching off the same location as it does in NS-1502, would retain the positioning required to interact with Arg-399 and Asn-202, whilst the flexibility of JN-02 allows the sulfonamide group to also interact with residues near Lys-263. The docking of JN-03 was performed for the purpose of considering all these interactions (Fig 8). For JN-03, as with the others, the phthalimide core is oriented next to Arg-20. The carboxyl group forms hydrogen bonds with Arg-399 and Asn-202, just as it does in NS-1502, a feature that was not prevalent in either JN-01 or JN-02. In place of the sulfonamide, the sulfate forms many hydrogen bonding interactions with the nearby residues including that of Tyr-74, Gln-118, Lys-263, and Arg-270.

It has been argued that the ability of sulfated estrogens to inhibit KAT is derived from a reversible reaction with the apoenzyme form of the protein, and it competes with PLP for the binding to the apoenzyme [37]. As mentioned previously, NS-1502, JN-01 and JN-02 displayed a similar competition with PLP to that seen for the estrogens, as they had reduced inhibition when PLP concentration increased. This work demonstrates that the potency of these types of inhibitors can be improved by introducing moieties, such as the sulfonamide or sulfate groups, to form stronger bonds within the active site and out-compete PLP for the apoenzyme. The notion that these moieties could be accommodated in the inhibitor binding region was proven to be correct by the assay results, although the docking of the compounds suggest that the intricate detail in which they bind can vary depending on the flexibility of each structure.

## Materials and methods

### General procedures

Commercially available reagents were used without additional purification unless otherwise stated. 4,5-dichlorophthalic anhydride, 4-aminobenzenesulfonamide, and 4-(2-aminoethyl)benzenesulfonamide were purchased from Sigma-Aldrich (Sydney, Australia). ^1^H-NMR (400 MHz) and ^13^C-NMR (101 MHz) spectroscopy was obtained using a Gemini 400-MR NMR spectrometer (Varian, Palo Alto, CA, USA), using DMSO-d_6_ as NMR solvent. Low-resolution ESI-MS was measured on an amaZon SL (Bruker Daltonics, Bremen, Germany). High resolution ESI-MS was performed on an Apex Ultra Fourier Transform Ion Cyclotron Resonance 7 T Mass Spectrometer (Bruker Daltonics, Bremen, Germany). The mass spectra were obtained by direct infusion electrospray ionization and are reported as mass to charge ratio (m/z) and relative intensity (%). The estimated purity for the synthesised inhibitors is > 93%, with the final yield approximately 70%. HPLC was performed using a CBM-20A Shimadzu HPLC system (Kyoto, Japan), with an Ascentis C18 HPLC column (particle size: 10 μm, length: 25 cm, internal diameter: 4.6 mm) purchased from Sigma-Aldrich (Sydney, Australia). The inhibition assay data was modelled with a nonlinear regression fit of the log[inhibitor] vs. normalised response (using a variable slope) in GraphPad Prism v7.02 software [40], and the IC_50_ is reported with 95% confidence intervals.

### Protein preparation

The recombinant KAT-II protein was expressed in *E*. *coli* Rosetta 2 cells with a hexa-histidine tag, and purified using nickel nitrilotriacetic acid column chromatography in our lab as previously described [41]. Recombinant KAT-I was expressed in *Spodoptera frugiperda* insect cells, precipitated using ammonium sulfate, and purified in a high salt ion exchange column as previously described [42].

### Synthesis of JN-01

A solution of 4,5-dichlorophthalic anhydride (2.5 mM) and 4-aminobenzenesulfonamide (2.5 mM) in glacial acetic acid (7 mL) was stirred and heated under reflux for 4 h. The product, 4-(5,6-dichloro-1,3-dioxo-1,3-dihydro-2H-isoindol-2-yl)benzenesulfonamide (JN-01), was recovered by precipitation using cold water and ethanol, filtered and dried. ^1^H-NMR (400 MHz, DMSO-d_6_): δ 8.33 (s, 2H), 7.98 (d, J = 8.7 Hz, 2H), 7.66 (d, J = 8.6 Hz, 2H), 7.48 (s, 2H). ^13^C-NMR (101 MHz, DMSO-d_6_): δ 125.69 (C-14,17), 126.43 (C-8,12), 127.49 (C-9,11), 131.55 (C-3,4), 134.44 (C-7), 137.66 (C-15,16), 143.51 (C-10), 164.91 (C-2,5) (S1 Appendix); HRESIMS: 424.97 m/z [C14H8N2O4S1Cl2 + CH3OH + Na]+ (Calcd. 424.974171) (S2 and S3 Appendices).

### Synthesis of JN-02

A solution of 4,5-dichlorophthalic anhydride (2.5 mM) and 4-(2-aminoethyl)benzenesulfonamide (2.5 mM) in glacial acetic acid (7 mL) was stirred and heated under reflux for 4 h. The product, 4-(5,6-dichloro-1,3-dioxo-1,3-dihydro-2H-isoindol-2-yl)ethyl benzenesulfonamide JN-02, was recovered by precipitation using cold water and ethanol, filtered and dried. ^1^H-NMR (400 MHz, DMSO-d_6_): δ 8.17 (s, 2H), 7.71 (d, J = 8.1 Hz, 2H), 7.40 (d, J = 8.1 Hz, 2H), 7.30 (s, 2H), 3.84 (t, J = 7.1 Hz, 2H), 2.99 (t, J = 7.1 Hz, 2H). ^13^C-NMR (101 MHz, DMSO-d_6_): δ 33.30 (C-8), 38.89 (C-7), 125.28 (C-16,19), 125.76 (C-11,13), 129.19 (C-10,14), 131.43 (C-3,4), 137.32 (C-17,18), 142.32 (C-12), 142.37 (C-9), 165.85 (C-2,5) (S4 Appendix).

### Inhibition studies using recombinant human KAT-I and KAT-II

0.5 μg of KAT-I or KAT-II was incubated at 37 °C for 10 min in a 50 μL reaction mixture containing 50 μM PLP, 5 mM α-ketoglutarate, 5 mM l-kynurenine in PBS, pH 7.4, with the inhibitor being studied (1–2000 μM) dissolved in DMSO. Equal volume of formic acid (0.8 M) was added to terminate the reaction, and 50 μL of this mixture was diluted to 1 mL. Kynurenine and KYNA produced during the reaction was analyzed by HPLC using a C18 reverse-phase column, with 50% (*v*/*v*) methanol and 50% (*v*/*v*) water used as the mobile phase, and with an injection volume of 20 μL. The column temperature was 20 °C. Kynurenine and KYNA were detected by UV detection at a wavelength of 330 nm. The retention time for kynurenine and KYNA were 2.5 mins and 3.5 mins, respectively.

### Docking of inhibitors into KAT-II

A crystal structure of human KAT-II (PDB ID: 2R2N [27]) was downloaded from the Protein Data Bank. 2R2N is a high resolution human KAT-II structure (1.95 Å) with the ligand, kynurenine, already occupying the active site. The PLP cofactor was removed from the active site of the protein as there was evidence it competed with the ligands in the inhibition studies. Optimisation and minimisation of the protein was performed using the Impref utility in IMPACT (Schrodinger LLC) and the OPLS-2005 [43] force field, and hydrogen atoms were also added and water molecules not involved in the reaction were removed, using the protein preparation wizard in Maestro version 10.4.017 (Schrodinger LLC). The active site was determined by the location of kynurenine in the crystal structure. The ligands were prepared using LigPrep [44], using the OPLS-2005 force field, and were docked using Glide [45] with XP docking [46]. Both enantiomers of NS-1502 and JN-03 were docked, with the best scoring pose represented in the figures.

## Conclusions

KAT-II inhibitors have been shown to reduce the production of KYNA, increase neurotransmitter release and improve memory tasks in animals [12, 13, 17]. Other compounds, particularly sulfate esters of estrogens, have also been shown to be very adept in inhibiting the KAT enzymes [37]. For sulfated estrogens, the 17-sulfate position appears to be very important in comparison to their unconjugated parent estrogens in this potency for inhibition, with docking also suggesting that it interacts with key active site residues [39].

Using the information derived from these estrogens, and the template of the previously designed reversible inhibitor NS-1502, we have synthesised two new inhibitors, JN-01 and JN-02. Notably, JN-01 and JN-02 differ from NS-1502 with the addition of a sulfonamide moiety, which seems to improve inhibition by approximately 3–4 fold, with an IC_50_ of 73.8 (± 3.9) μM and 112.8 (± 6.1) μM, respectively. This finding suggests that knowledge gleaned from sulfated estrogens binding can help improve the potency of current inhibitors. A sulfate (or like-minded) moiety can be placed to mimic the interactions of the 17-sulfate in estradiol disulfate, and greatly improve potential inhibition.

## Supporting information

S1 Fig Structures of existing KAT-II inhibitors. (TIF)

S2 Fig Structure of estradiol disulfate. (TIF)

S3 Fig Estradiol disulfate docked into the KAT-II active site. The amino acids within 5.0 Å of estradiol disulfate (green) were chosen for display. Residues Tyr-142 and Gly-144 were removed for clarity. The 3-sulfate forms hydrogen bonds (yellow dashes) with Arg-20 and the 17-sulfate forms hydrogen bonds with Asn-202 and Lys-263. Image generated with PyMOL [29].
(TIF)

S1 Appendix ^1^H-NMR and ^13^C-NMR results for JN-01. (PDF)

S2 Appendix HRESIMS results for JN-01. (PDF)

S3 Appendix HRESIMS results for JN-01. (PDF)

S4 Appendix ^1^H-NMR and ^13^C-NMR results for JN-02. (PDF)
