# Supplementary figures and images for: Improvement of kynurenine aminotransferase-II inhibitors guided by mimicking sulfate esters
Source: PLoS One. 2018 Apr 24;13(4):e0196404. doi: 10.1371/journal.pone.0196404 (PMC5915280; doi:10.1371/journal.pone.0196404)

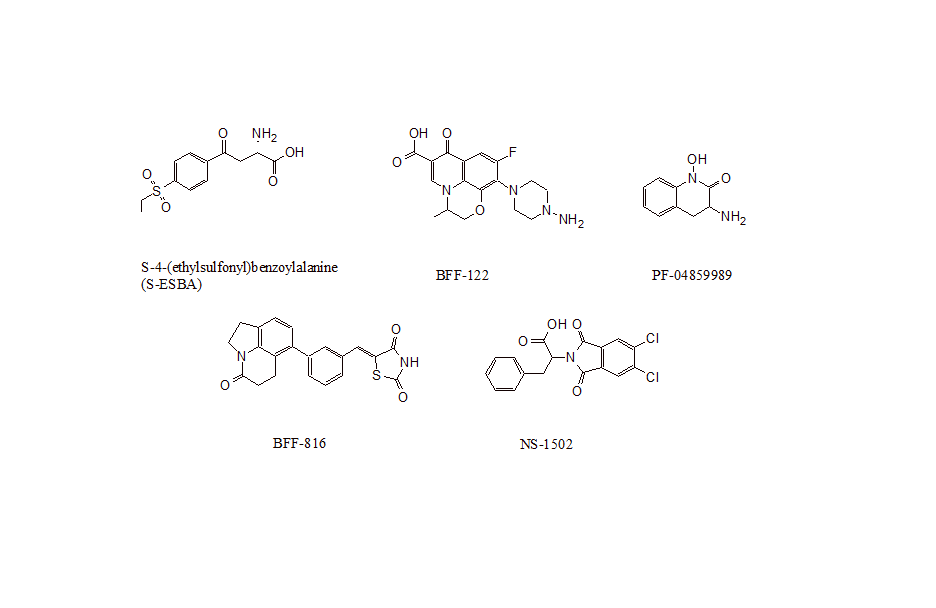

Supplement: S1 Fig — (TIF) [file pone.0196404.s001.tif]

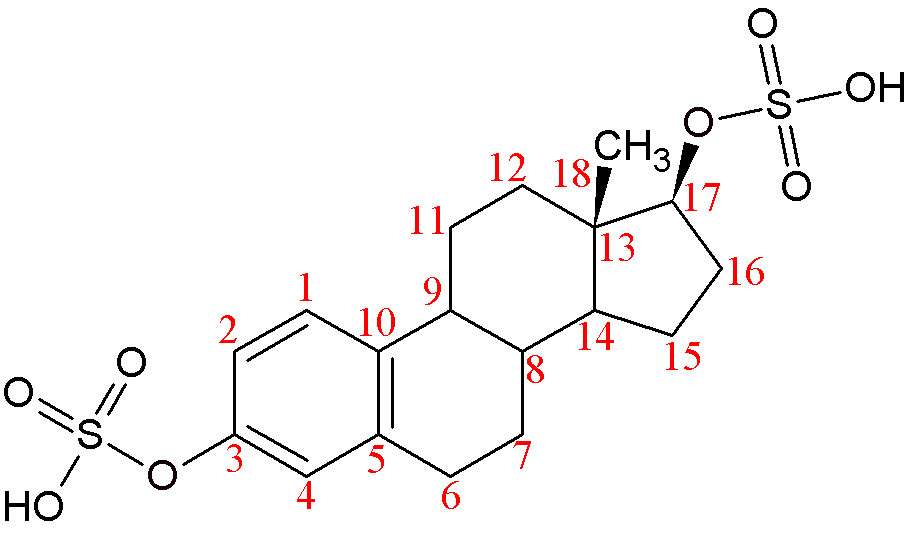

Supplement: S2 Fig — (TIF) [file pone.0196404.s002.tif]

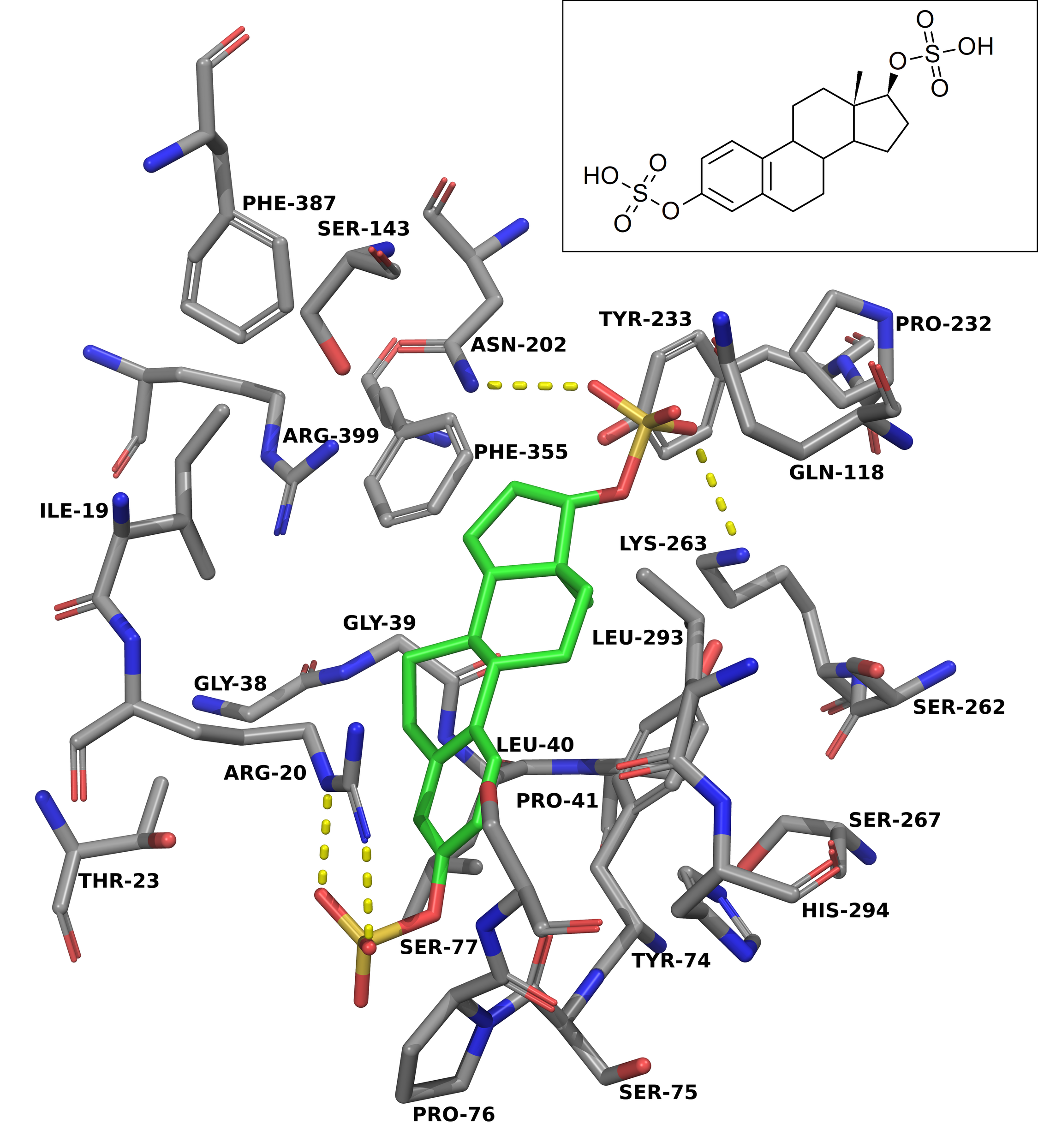

Supplement: S3 Fig — The amino acids within 5.0 Å of estradiol disulfate (green) were chosen for display. Residues Tyr-142 and Gly-144 were removed for clarity. The 3-sulfate forms hydrogen bonds (yellow dashes) with Arg-20 and the 17-sulfate forms hydrogen bonds with Asn-202 and Lys-263. Image generated with PyMOL [29]. (TIF) [file pone.0196404.s003.tif]

$^1\text{H}$  NMR (400 MHz,  $\text{DMSO-}d_6$ )  $\delta$  8.33 (s, 2H), 7.98 (d,  $J = 8.7$  Hz, 2H), 7.66 (d,  $J = 8.6$  Hz, 2H), 7.48 (s, 2H).

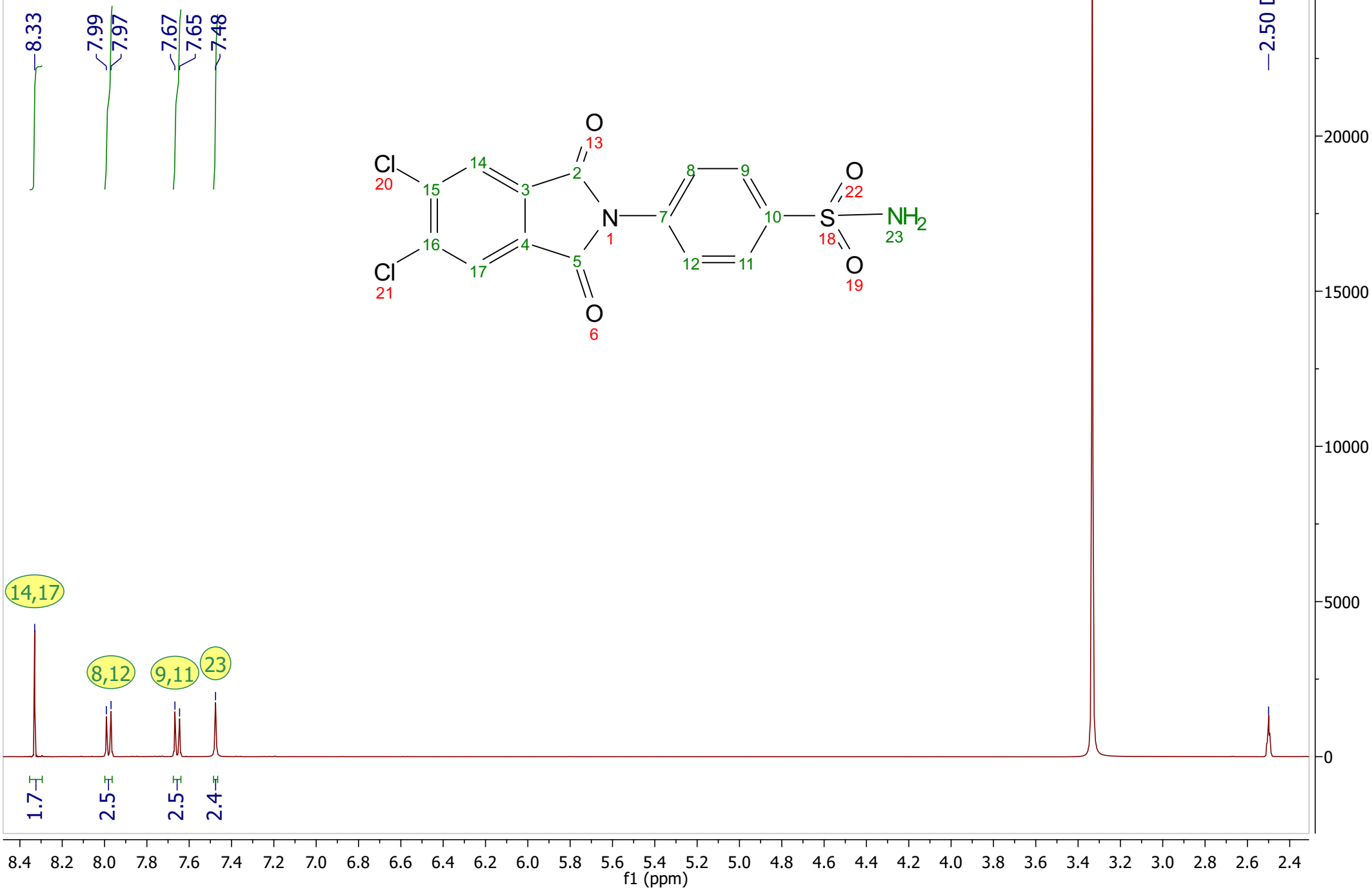

$^{13}\text{C}$  NMR (101 MHz, dms $\text{o}$ )  $\delta$  164.91, 143.51, 137.66, 134.44, 131.55, 127.49, 126.43, 125.69, 39.52.

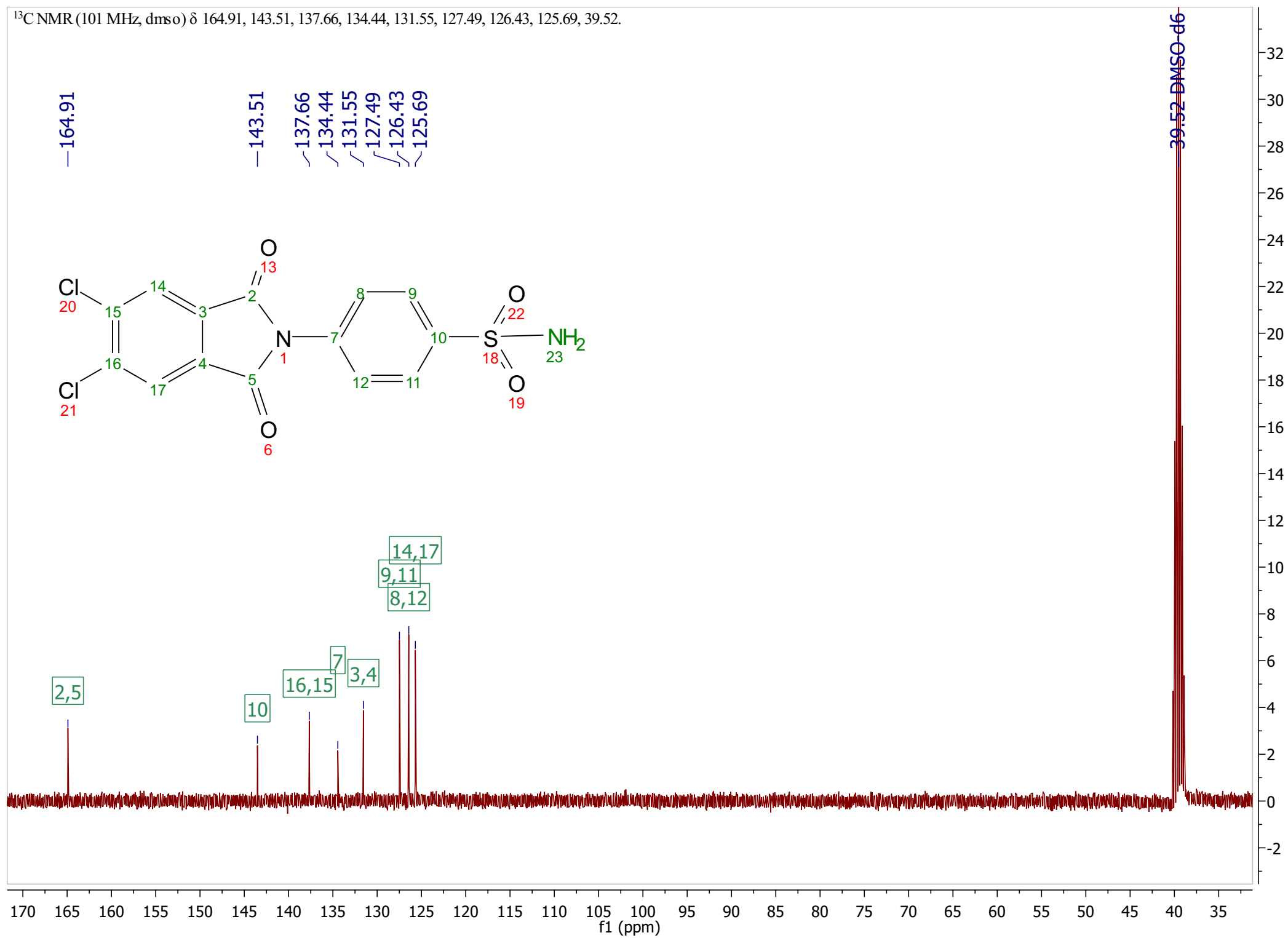

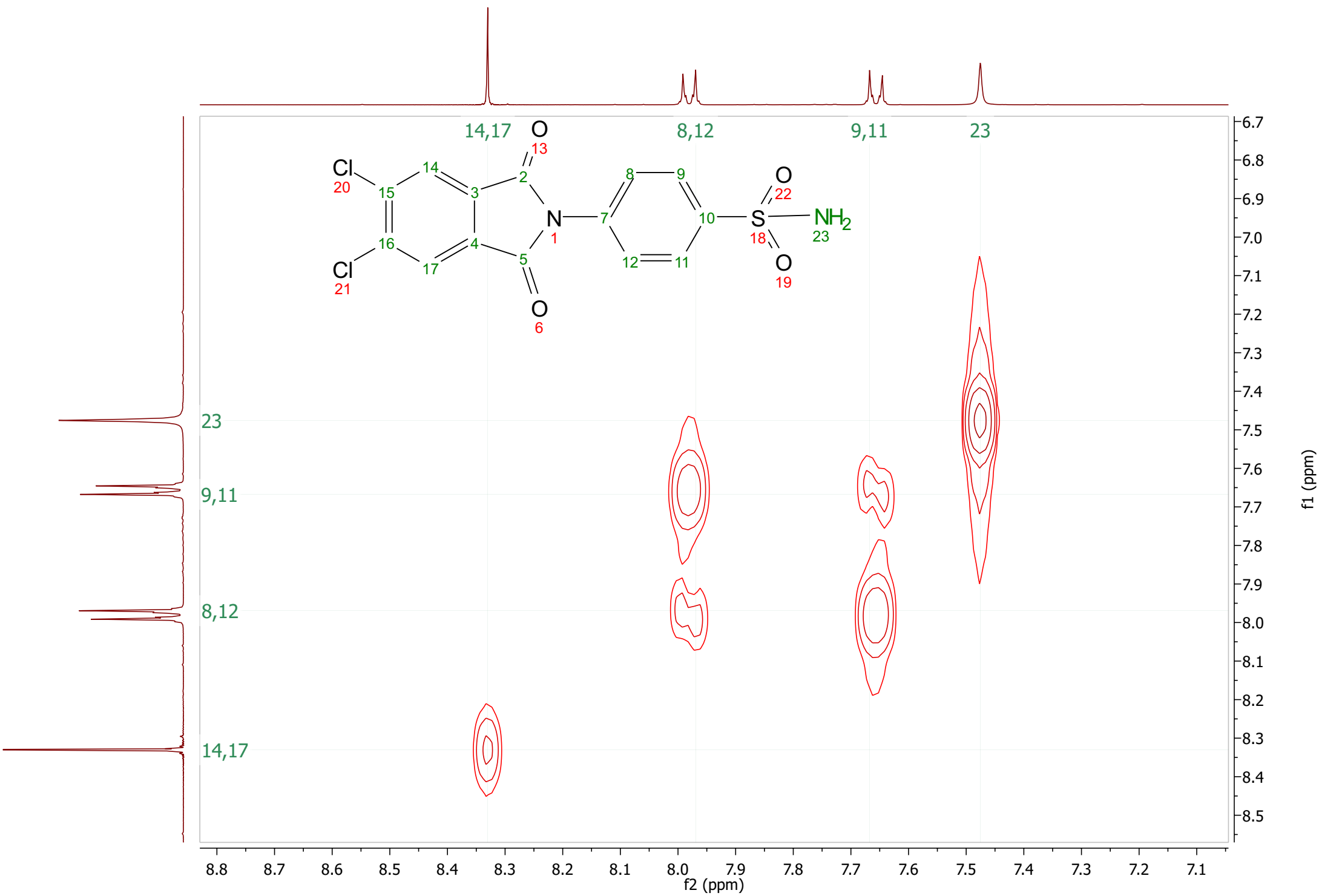

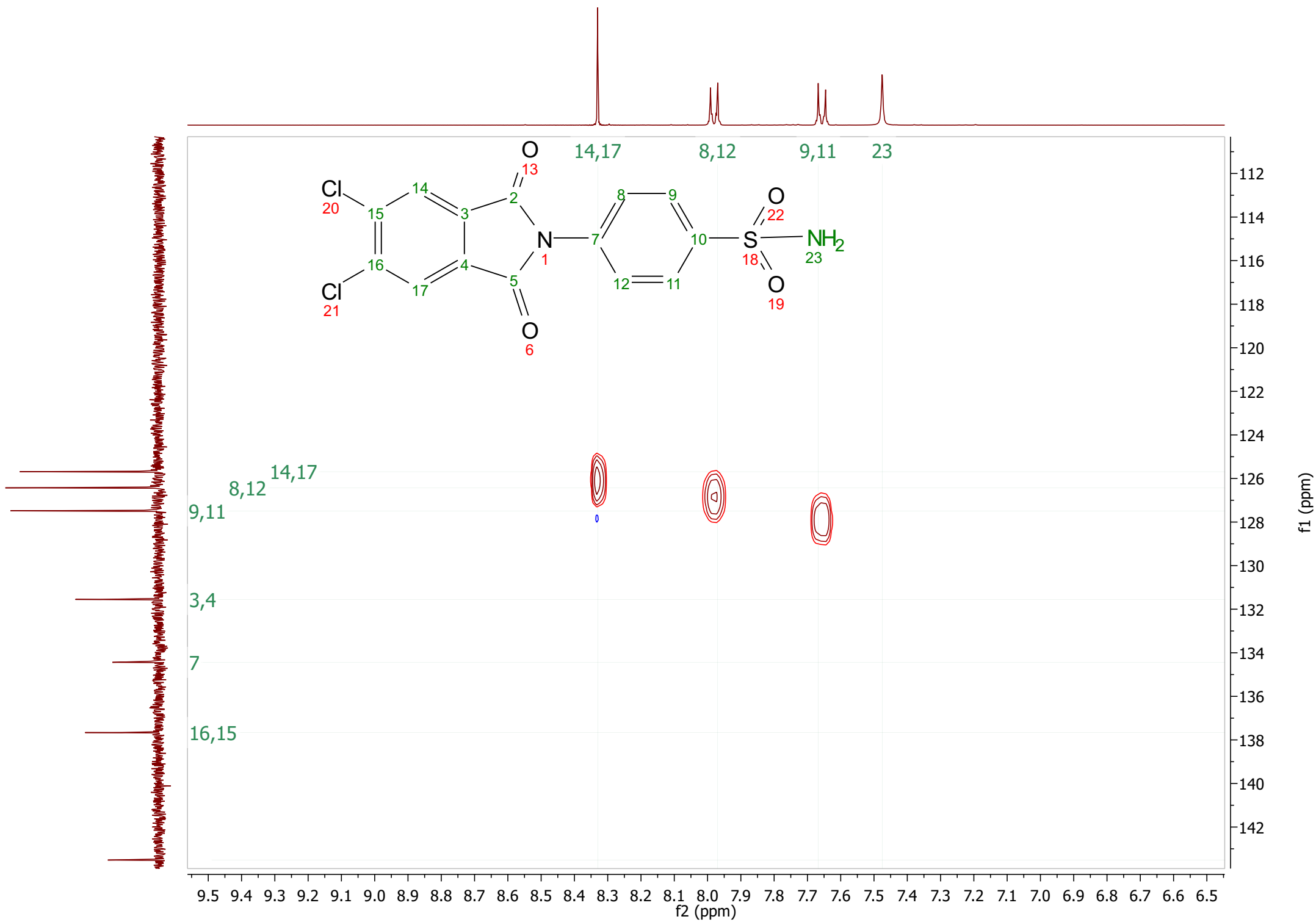

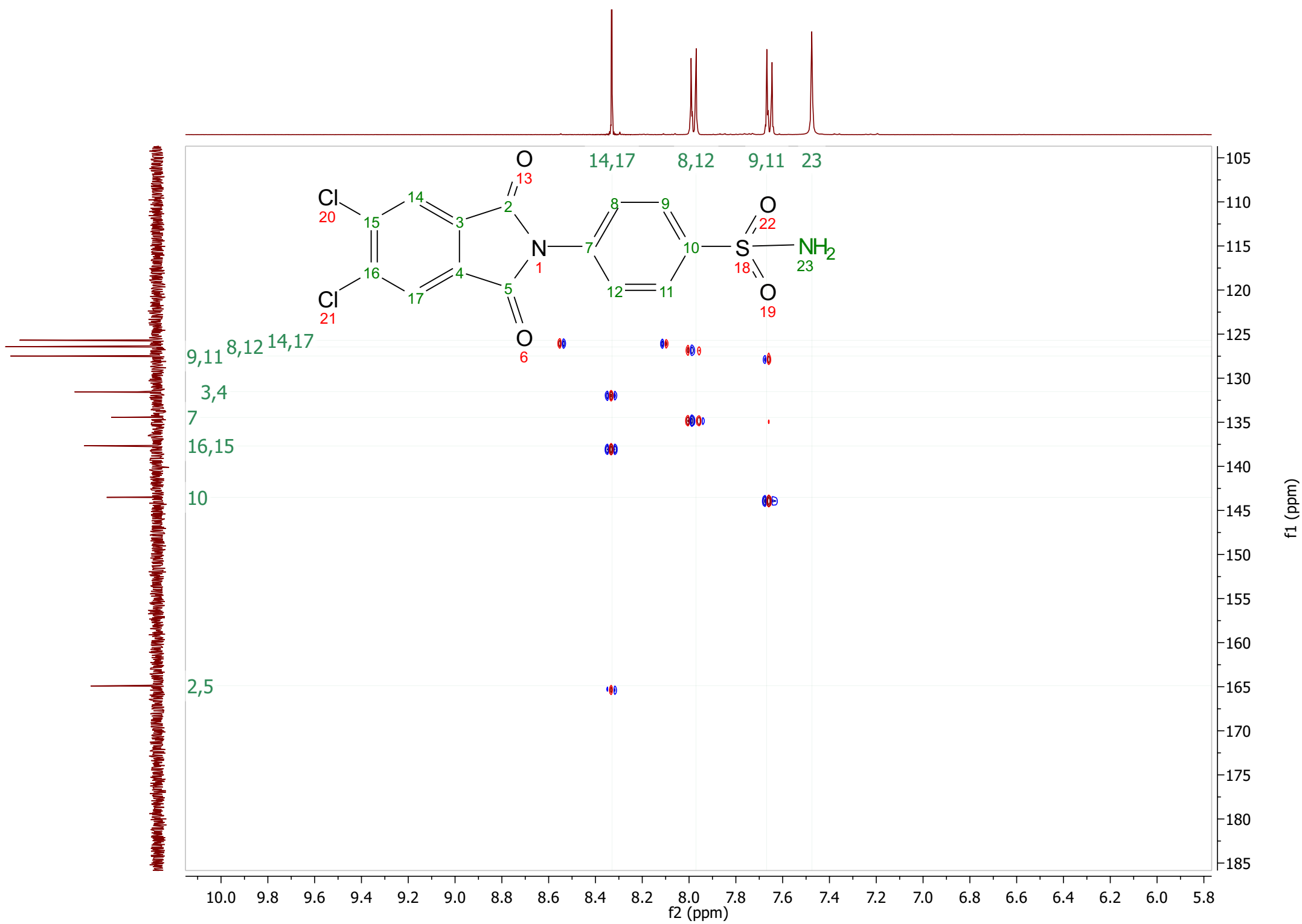

Supplement: S1 Appendix — (PDF) [file pone.0196404.s004.pdf]

$^1\text{H}$  NMR (400 MHz,  $\text{DMSO-}d_6$ )  $\delta$  8.17 (s, 2H), 7.71 (d,  $J = 8.1$  Hz, 2H), 7.40 (d,  $J = 8.1$  Hz, 2H), 7.30 (s, 2H), 3.84 (t,  $J = 7.1$  Hz, 2H), 2.99 (t,  $J = 7.1$  Hz, 2H).

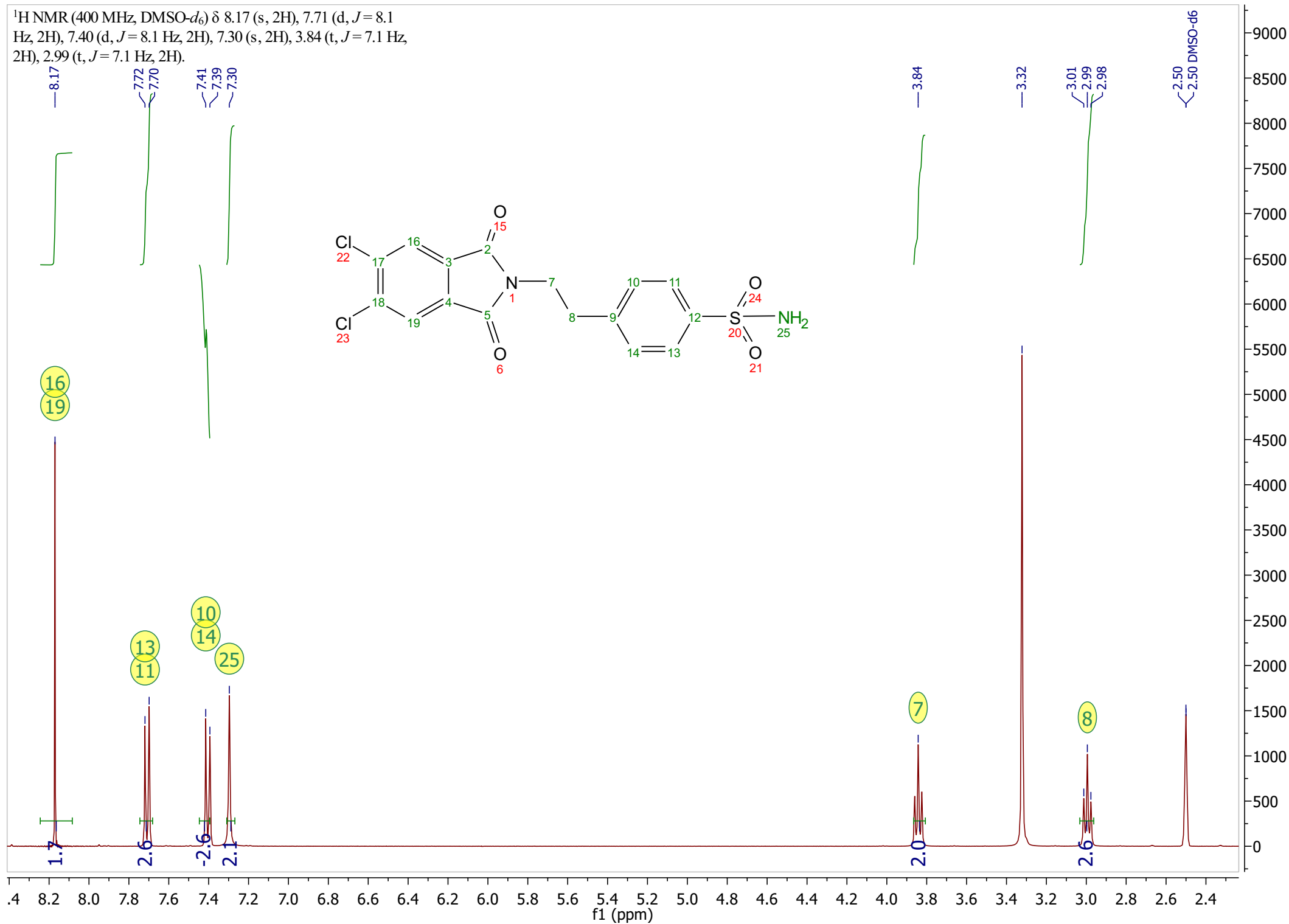

$^{13}\text{C}$  NMR (101 MHz, dms $\text{o}$ )  $\delta$  165.85, 142.37, 142.32, 137.32, 131.43, 129.19, 125.76, 125.28, 39.52, 38.89, 33.30.

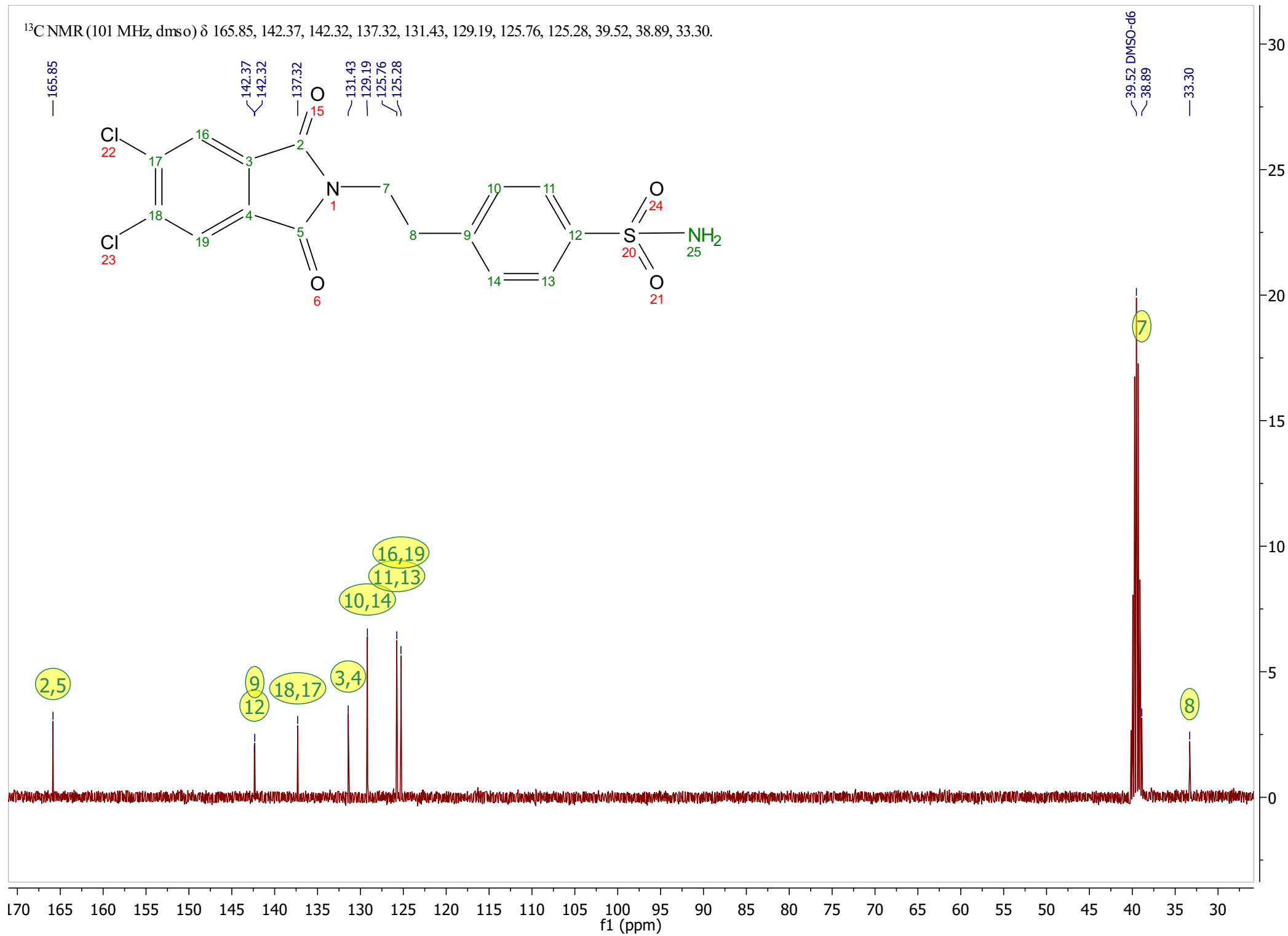

Supplement: S4 Appendix — (PDF) [file pone.0196404.s007.pdf]
