# Supplementary material for: Improvement of kynurenine aminotransferase-II inhibitors guided by mimicking sulfate esters
Source: PLoS One. 2018 Apr 24;13(4):e0196404. doi: 10.1371/journal.pone.0196404 (PMC5915280; doi:10.1371/journal.pone.0196404)

Generic Display Report

Analysis Info

Analysis Name	D:\Data\Nick-2017-files\ESI_Positive\10-March\2017-03-10-posesi-service_000007.d	Acquisition Date	10/03/2017 10:20:52 AM
Method	1MW Positive ESI	Operator	
Sample Name	GN01	Instrument	apex-Ultra
Comment	ACN 1M TOF delay 0.0007s, Q1 300 m/z		

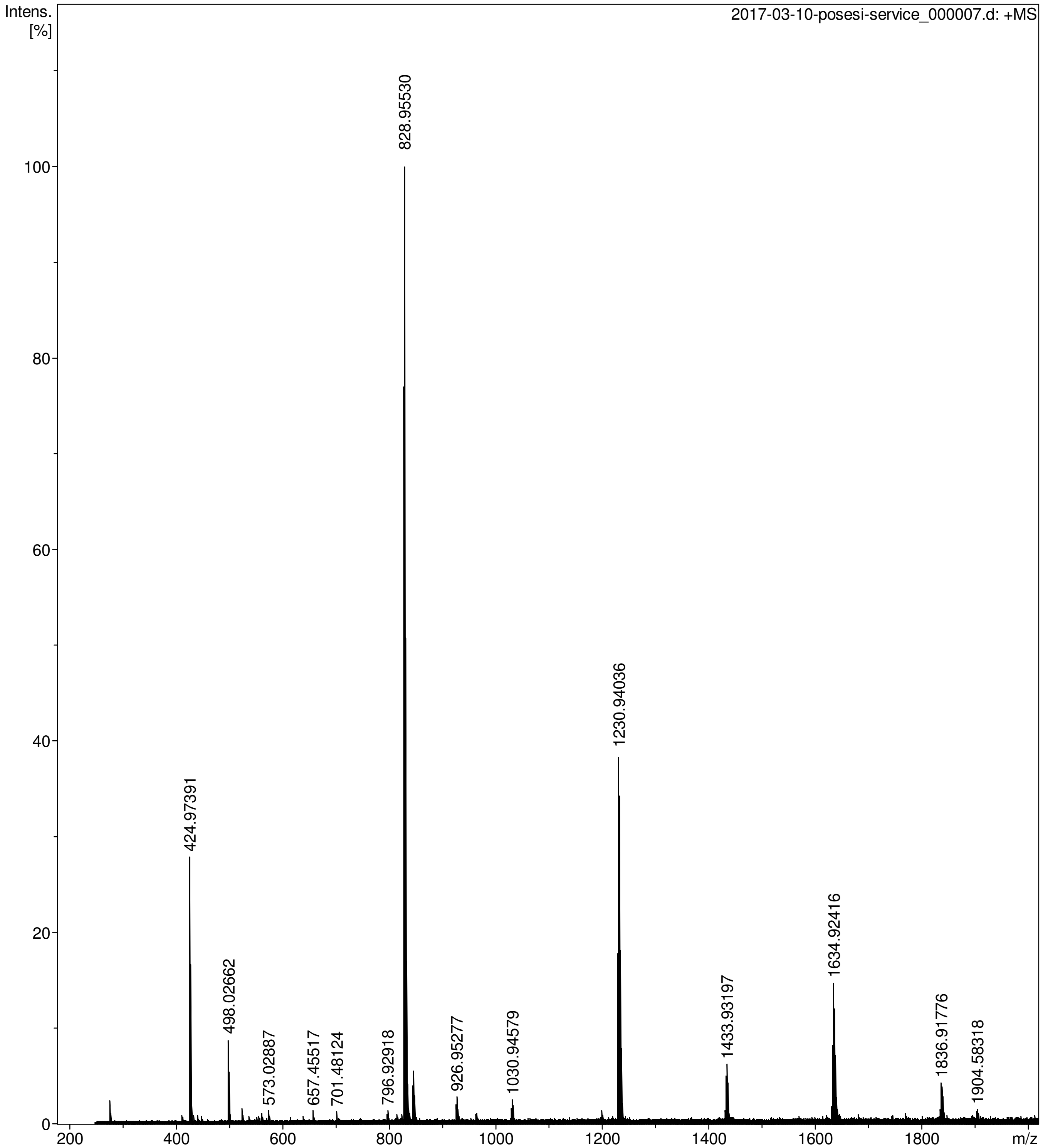

Supplement: S2 Appendix — (PDF) [file pone.0196404.s005.pdf]
